# COMPLICATED PNEUMONIA WITH EMPYEMA CAUSED BY STREPTOCOCCUS ANGINOSUS IN A CHILD

**DOI:** 10.1590/1984-0462/2020/38/2018258

**Published:** 2020-03-09

**Authors:** Ana Reis-Melo, Diana Soares, Manuel Ferreira Magalhães, Catarina Ferraz, Luísa Vaz

**Affiliations:** a Centro Hospitalar de São João, Porto, Portugal.; b Centro Hospitalar Vila Nova de Gaia, Gaia, Portugal.

**Keywords:** Bacterial pneumonia, Pleural empyema, Streptococcus anginosus, Video-assisted thoracic surgery, Pneumonia bacteriana, Empiema pleural, Streptococcus anginosus, Cirurgia torácica por videotoracoscopia

## Abstract

**Objective:**

To highlight the pathogenicity of *Streptococcus anginosus*, which is rare in pediatric patients, but can cause severe infections that are known to have a better outcome when treated early with interventional procedures and prolonged antibiotic therapy.

**Case:**

**description:** The patient is a 6-year-old boy with global developmental delay, examined in the emergency room due to fever and respiratory distress. The physical examination and diagnostic workout revealed complicated pneumonia with empyema of the left hemithorax; he started antibiotic therapy and underwent thoracic drainage. Pleural fluid cultures grew *Streptococcus anginosus*. On day 11, the child had a clinical deterioration with recurrence of fever, hypoxia, and respiratory distress. At this point, considering the causative agent, he was submitted to video-assisted thoracoscopic decortication, with good progress thereafter.

**Comments:**

*Streptococcus anginosus* is a commensal bacterium of the human oral cavity capable of causing severe systemic infections. Although reports of complicated thoracic infections with this agent are rare in the pediatric population, they have been increasing in adults. *Streptococcus anginosus* has a high capacity to form abscess and empyema, requiring different therapeutic approaches when compared to complicated pneumonia caused by other agents.

## RESUMO

**Objetivo:**

Alertar para a patogenicidade do *Streptococcus anginosus* que, apesar de raro em pediatria, pode causar infeções graves que necessitam de tratamento invasivo e antibioterapia de longo curso para obter um melhor prognóstico.

**Descrição:**

**do caso:** Criança de seis anos, com atraso do desenvolvimento psicomotor, avaliado no serviço de urgência por febre e dificuldade respiratória. O exame físico, juntamente com os exames complementares, revelou uma pneumonia complicada com empiema no hemitórax esquerdo, tendo iniciado antibioterapia e sido submetido à drenagem do líquido pleural. Foi identificado *Streptococcus anginosus* nesse líquido*.* No 11º dia de doença, a criança agravou o seu estado clínico, com recidiva da febre, hipoxemia e dificuldade respiratória. Considerando-se o microrganismo identificado, o paciente foi submetido à decorticação pulmonar por videotoracoscopia, com boa evolução clínica posterior.

**Comentários:**

*Streptococcus anginosus* é uma bactéria comensal da cavidade oral humana, que pode causar infecções sistêmicas graves. Apesar de serem raros os casos descritos em pediatria, têm sido cada vez mais descritas infecções torácicas complicadas em adultos. Esse microrganismo também tem a capacidade de formar abcessos e empiemas, que precisam de intervenções terapêuticas diferentes, quando comparados a pneumonias complicadas causadas por outros agentes.

## INTRODUCTION

*Streptococcus anginosus* is a gram-positive, facultative anaerobic bacterium related to *Streptococcus constellatus* and *Streptococcus intermedius.* Together, these species constitute the *Streptococcus anginosus* group (SAG), formerly known as the *Streptococcus milleri* group.^1^ They may cause pyogenic infections that usually require prolonged antibiotic therapy and surgical interventions.^2^ ^,^ ^3^ ^,^ ^4^ All 3 are commensal bacteria, but recent evidence shows that they are also pathogenic and may cause abscesses or systemic infections.^5^ They are associated with abdominal, central nervous system, and pleuropulmonary infections;^6^ ^,^ ^7^ *S. anginosus* is often found in blood cultures with or without identification of the primary site of infection.^5^ ^,^ ^8^ Importantly, while *S. anginosus* is increasingly recognized as a significant cause of pulmonary infections in adults, studies reporting its identification in the pediatric population are rare.

## CASE DESCRIPTION

A 6-year-old male with early infantile encephalopathy, global developmental delay, and recurrent respiratory infections since the first year of life, visited the emergency room of a community hospital with low-grade fever, irritability, and grunting on day 1 of the disease. During the physical examination, the patient presented skin pallor, 88% peripheral oxygen saturation, signs of respiratory distress, and pulmonary auscultation with diminished respiratory sounds and crackles on the lower half of the left hemithorax. Laboratory findings included: hemoglobin 11.0 g/dL, white blood cell count 12,100/uL with 9.8% lymphocytes and 71.6% neutrophils, platelets 640,000/uL, and C-reactive protein (CRP) 103 mg/L. The chest X-ray showed hypolucency on the left hemithorax. The patient was admitted and initiated amoxicillin plus clavulanic acid 50 mg/kg/day, twice a day, intravenously. On day 2, multiloculated pleural effusion was detected with thoracic ultrasound. Next, the child underwent a left thoracic drainage with drain placement; the pleural fluid was macroscopically purulent, and the laboratory analysis revealed a fluid with 62,700 cells/mm^3^ (85% neutrophils), pH 6.5, glucose <5 mg/dL, total protein 5 g/dL, cholesterol 80 mg/dL, triglycerides 36 mg/dL, adenosine deaminase 45 U/L, and lactate dehydrogenase 1,440 U/L; cultures were also requested. At this stage, antibiotic therapy was changed to ceftriaxone 80 mg/kg/day. In the following 48 hours, the patient had clinical improvement, without fever or respiratory distress. The bacteriological examination of the pleuritic fluid identified *Streptococcus anginosus*, sensitive to penicillin and cefotaxime; blood cultures were sterile.

**Figure 1:**
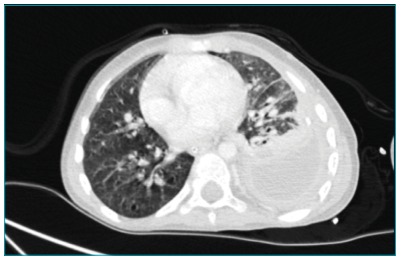
Thoracic computed tomography scan performed on admission to a tertiary hospital, showing loculated pleural effusion on the left hemithorax, measuring 6.7 × 3.2 cm, with thickening and high uptake of pleural layers. Areas of consolidation and atelectasis are also visible.

On day 11, the child had a recurrence of fever, and the left pulmonary sounds ceased. At this point, the child was transferred to a tertiary hospital, and his blood test revealed leukocytosis (38,000/uL) with neutrophilia (81.7%), thrombocytosis (843,000/uL), and elevated CRP (122 mg/L). He had an ill-appearance, severe signs of respiratory distress, and hypoxemia. Thoracic computed tomography scan showed left empyema, atelectasis, and consolidation with necrosis on the lower pulmonary left lobe (Figure 1). Abdominal abscesses and endocarditis were excluded by abdominal ultrasound and echocardiogram. Clindamycin (30 mg/kg/day, 4x a day, intravenously) was added to the therapy for anaerobic coverage as the clinical state of the child was severe.

**Figure 2:**
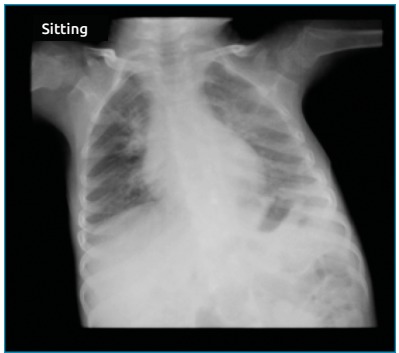
Chest X-ray performed during the follow-up with no abnormalities.

On day 12, the patient underwent a left decortication via video-assisted thoracoscopic surgery, which revealed large fibrin deposits and adhesions between the lung and the pleura; two thoracic drains were placed and kept for eight days. The patient showed significant clinical improvement in the first 24 hours after surgery. Pulmonary sounds gradually improved, and he had a full recovery after a four-week course of ceftriaxone and a two-week course of clindamycin. In the follow-up one and three weeks after discharge (Figure 2), the child was well, and the chest X-ray was normal.

## DISCUSSION

The SAG is presently recognized as an important pathogenic group and not only as commensal contaminants on cultures from biological products. This recent acknowledgment may be due to advances in microbiological techniques that have higher sensitivity and are able to identify SAG in infectious diseases.^5^ In the past few years, some studies reported more cases of invasive infections caused by SAG in adults; *S. anginosus* was the most predominant species.^5^ ^,^ ^9^ In the pediatric population, there is a lack of case reports focusing on this microorganism, which is a commonly underappreciated pathogen. A recent series identified abscesses caused by *S. intermedius* in 48 children for seven years. Among them, 40% had a complicated and/or life-threatening illness, which illustrates the morbidity associated with this pathogen.^1^ Another pediatric series showed no *S. anginosus* among infections caused by SAG,^10^ demonstrating the clinical discrepancy in the literature. Also, in some reported cases, the causative microorganism belongs to SAG, but they do not identify the member within the group, which makes it difficult to clearly determine the individual SAG species among children and its association with the infection site.

*S. anginosus* is a capsulated bacterium present in the oral cavity, gastrointestinal mucosa, and genitourinary tract. Virulence factors are yet to be established,^5^ but adhesins play an important role in its increased ability to adhere to buccal epithelial cells and cause infections. Other virulence factors include ß-hemolysins and hydrogen sulfide. In our case, the mechanism of infection was probably the aspiration of the commensal agent located in the oropharynges.

The three SAG pathogens have different clinical presentations - *Streptococcus anginosus*, *Streptococcus constellatus*, and *Streptococcus intermedius*. *S. anginosus* is more commonly isolated in blood cultures, and the other two have a higher capacity to form abscesses.^5^ ^,^ ^11^ Previous studies in the pediatric age group reported brain abscesses or intracranial complications^12^ and intra-abdominal infections^10^ caused by SAG pathogens. Older adults are more affected by thoracic infections, particularly pneumonia, caused by *S. anginosus*.^13^ In pleuropulmonary infections, *S. anginosus* appears to have the capacity to cross tissue planes, often leading to the formation of empyema that extends to the soft tissue adjacent to the parietal pleura, which was not the case in this child.^11^ In a pediatric series, complex empyema and pulmonary abscess caused by SAG were the most prevalent conditions, corroborating the pyogenic capacity of these bacteria; all patients were immunocompetent and needed at least one interventional procedure. In the first 24 hours of the disease, our patient presented empyema, demonstrating the highly pathogenic and abscess formation capacity related to *S. anginosus*. The child only showed good clinical and radiological evolution after a thoracoscopic intervention and despite appropriate antibiotic therapy, demonstrating the importance of surgery in these cases.^6^ ^,^ ^14^ ^,^ ^15^ Empyema in the fibrinopurulent or organizing stage should be considered an indication for surgical management.^16^ The antibiotic course is usually long, with a mean duration of up to 34 days, and most SAG isolates seem to be sensitive to penicillin and cefotaxime,^10^ ^,^ ^17^ ^,^ ^18^ like in our case report. Some reports indicate that patients infected by *S. anginosus* can be co-infected by other agents, including anaerobes, so clindamycin was added at the begin of the treatment. Among the SAG, *S. anginosus* is the most commonly implicated in infective endocarditis, with elevated mortality,^5^ ^,^ ^19^ which was excluded in our case.

In recent years, the incidence of empyema has increased; meanwhile, *S. anginosus* has been more recognized as a frequent cause among adults.^20^ Our case appears to be one of the very few published cases reporting a pneumonia caused by *S. anginosus* in the pediatric age group; the only other similar case found was a report of a Chilean child.^18^ However, the authors found this difficult to confirm, as the term SAG is usually used instead of the actual member of the group. Also, this agent is often responsible for other types of infection in these ages. Our finding highlights the importance of differentiating the three organisms that form SAG in order to understand their unique pathogenicity better. The use of pneumococcal conjugate vaccine could change the agents causing complicated pneumonia. Pediatricians should be aware of these emerging agents. *S. anginosus* can cause severe infections that require rapid diagnosis and appropriate treatment to reduce the associated morbidity and mortality.^9^ ^,^ ^15^
